# Effects of Antiseptic Formulations on Oral Microbiota and Related Systemic Diseases: A Scoping Review

**DOI:** 10.3390/antibiotics14080815

**Published:** 2025-08-08

**Authors:** Angela Angjelova, Elena Jovanova, Alessandro Polizzi, Rosalia Leonardi, Gaetano Isola

**Affiliations:** 1 Unit of Periodontology, Department of General Surgery and Surgical-Medical Specialties, School of Dentistry, University of Catania, 95123 Catania, Italy; angela.angjelova@students.stomfak.ukim.mk (A.A.); elena.jovanova@students.stomfak.ukim.mk (E.J.); rleonard@unict.it (R.L.); gaetano.isola@unict.it (G.I.); 2 International Research Center on Periodontal and Systemic Health “PerioHealth”, University of Catania, 95123 Catania, Italy

**Keywords:** oral health, oral microbiota, antiseptic formulations, dysbiosis, systemic health

## Abstract

**Background:** Oral antiseptic formulations are widely used as adjuncts in oral hygiene to reduce pathogenic microorganisms and prevent oral diseases. While these agents are effective in controlling biofilm, their broader effects may disrupt the oral microbiota’s balance, potentially contributing to systemic health implications. The complex relationship between antiseptic use, microbial composition, and systemic outcomes remains insufficiently mapped. **Objective:** This scoping review aimed to explore and map the current evidence regarding the impact of antiseptic formulations on oral microbiota composition and to examine their potential associations with systemic diseases. **Methods:** A comprehensive literature search was performed using PubMed, Scopus, and Web of Science up to June 2025. Studies were included if they investigated antiseptic formulations commonly used in oral healthcare—such as chlorhexidine, essential oils, and cetylpyridinium chloride—and reported effects on oral microbiota and/or systemic health. Eligible study types included human clinical trials, observational studies, in vitro, and animal studies. Two reviewers independently screened and selected studies, with disagreements resolved by consensus. Data extraction focused on study design, antiseptic agents, microbial outcomes, and systemic implications. A total of 12 studies were included and charted. **Results:** The included studies demonstrated that oral antiseptics effectively reduce pathogenic microorganisms and improve clinical outcomes in oral diseases such as gingivitis and periodontitis. However, several studies also reported alterations in commensal microbial communities, suggesting a potential for dysbiosis. Some studies indicated possible links between antiseptic-induced microbial changes and systemic conditions, including cardiovascular and respiratory diseases. **Conclusions:** The evidence highlights a dual effect of antiseptic formulations: while beneficial in controlling oral pathogens, they may disrupt microbial homeostasis with possible systemic consequences. Further research is needed to evaluate long-term effects and develop targeted, microbiota-preserving oral hygiene strategies.

## 1. Introduction

Oral health is a critical component of overall systemic well-being, with the oral cavity serving as a key interface between the external environment and the body’s internal system [1]. The oral microbiome comprises diverse microbial communities, including bacteria, viruses, protozoa, fungi, and archaea that are important in maintaining both oral and systemic health [2]. Approximately 700 microbial species inhabit the oral cavity, of which around 54% have been successfully cultured and classified, as documented in the Human Oral Microbiome Database [3]. These include commonly identified genera and species such as *Streptococcus mutans*, *Porphyromonas gingivalis*, *Fusobacterium nucleatum*, *Actinomyces naeslundii*, and *Veillonella parvula*, which play key roles in both oral health and disease [4].

The oral microbiome typically exists as a biofilm that adheres to oral surfaces. Its formation begins with salivary pellicle deposition, followed by initial colonization by commensals, and subsequent microbial succession leads to a complex, multispecies structure. This organization supports oral homeostasis, defending the oral cavity, and inhibiting the progression of diseases [5]. Through its innate defence mechanisms, it acts as a protective barrier [1] by reducing the colonization of harmful pathogens and preventing potential infections and diseases in the oral cavity—such as dental caries and periodontal disease—by controlling the growth and activity of pathogenic bacteria [1,6,7].

As the oral cavity is the entry point to the body, the oral microbiome is continuously altered by various factors, including mechanical forces, food intake, oral hygiene practices, and lifestyle behaviours such as smoking, vaping, and alcohol intake [8]. These environmental and host-related factors can disrupt the ecological balance of the oral microbiome, triggering a shift from symbiosis to dysbiosis. Therefore, the resulting microbial imbalance may favor the proliferation of pathogenic species and impair immune regulation, thereby promoting oral infections such as dental caries and periodontitis [9]. Oral dysbiosis—apart from its involvement in oral diseases—induces a localized inflammation in the oral cavity, which may potentially contribute to the persistence of chronic low-grade systemic inflammation [10] and represents a significant risk factor for the development of systemic disorders, including bacterial endocarditis, diabetes, cancer, cardiovascular diseases (CVD), respiratory diseases, rheumatoid arthritis (RA), and adverse pregnancy outcomes [10,11]. The vascular mechanisms underlying these associations include (1) systemic inflammation by the cytokine release into the bloodstream, (2) bacteriemia, (3) oral bacterial enterosalivary pathway, and (4) genetic susceptibility [12].

Oral antiseptic formulations are widely recognized in oral healthcare practice for their clinical benefits in reducing oral biofilm formation [13], used independently or as adjuncts to mechanical oral hygiene methods. While they provide local benefits, they correspondingly may be associated with an increased risk or aggravation of systemic conditions [12]. Restoring and maintaining oral health, therefore, requires a more comprehensive and biologically informed approach. Mechanical plaque removal remains the cornerstone of oral hygiene, although it is increasingly complemented by targeted interventions aimed at modulating the oral microbiome rather than eradicating it. Together, these approaches aim to restore and maintain a healthy oral microbiome while minimizing disruption and potential side effects [14].

This scoping review aims to map the current evidence on the effects of antiseptic formulations on oral microbiota composition and their potential systemic implications. Using the Population, Concept, and Context (PCC) framework, this review identifies key themes, knowledge gaps, and directions for future research, emphasizing the importance of personalized oral hygiene strategies that preserve microbial balance and support overall systemic health.

## 2. Materials and Methods

This scoping review was conducted following the methodological guidelines of the Joanna Briggs Institute (JBI) and is reported following the PRISMA-ScR (Preferred Reporting Items for Systematic reviews and Meta-Analyses extension for Scoping Reviews) checklist (see Supplementary Materials).

### 2.1. Objective and PCC Framework

This scoping review aimed to map existing evidence on the effects of antiseptic formulations on oral microbiota composition and dynamics, and their potential systemic health implications.

This review was guided by the Population, Concept, and Context (PCC) framework:

- Population (P): Human subjects, in vitro, and animal models related to oral health;
- Concept (C): Use of antiseptic formulations (e.g., chlorhexidine, essential oils, and cetylpyridinium chloride) and their impact on oral microbiota and systemic health;
- Context (C): Clinical and experimental settings involving oral hygiene and systemic implications.

### 2.2. Research Question

The primary research question guiding this review was as follows: What is the impact of antiseptic formulations on the composition and function of oral microbiota, and what systemic consequences may be associated with their use?

### 2.3. Eligibility Criteria

Studies were included in this review based on the following criteria: (1) studies investigating the effects of antiseptic formulations commonly used in oral healthcare (e.g., chlorhexidine, essential oils, and cetylpyridinium chloride) on oral microbiota or systemic health; (2) human in vivo studies; (3) in vitro or animal studies; (4) clinical trials and observational studies; (5) articles published in English.

Exclusion criteria were as follows: (1) studies focusing on antiseptic use for non-oral conditions; (2) studies not available in English; (3) reviews.

### 2.4. Information Source and Search Strategy

An electronic literature search was conducted using PubMed, Scopus, and Web of Science databases up to and including June 2025. The search strategy combined keywords and MeSH terms related to “oral antiseptics,” “oral microbiota,” “dysbiosis,” “systemic diseases,” and “oral health,” utilizing Boolean operators (AND, OR) to refine the search results according to each database’s syntax rules.

### 2.5. Study Selection

**Figure 1:**
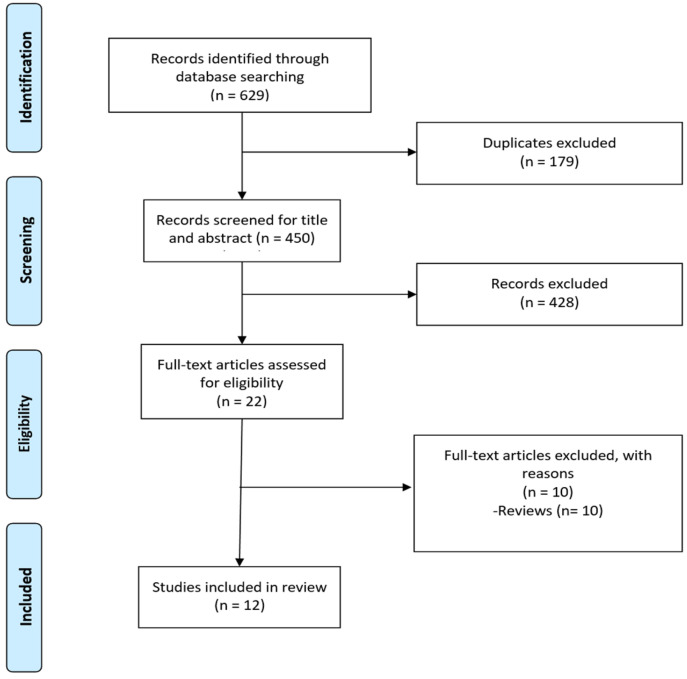
PRISMA flowchart showing the selection process for included studies.

Two independent reviewers screened titles and abstracts for eligibility according to the predefined criteria. Full texts of potentially relevant studies were then assessed for inclusion. Discrepancies were resolved by consensus or consultation with a third reviewer. A PRISMA-ScR flow diagram (Figure 1) illustrates the screening and selection process.

### 2.6. Data Charting and Extraction

Data extracted from the included studies comprised the following: (a) general study characteristics (author, year, country, and study design); (b) aim of the study; (c) antiseptic formulation(s) investigated; (d) main outcomes; (e) key conclusions.

The microbial species discussed and tabulated in this review were selected based on their recognized roles as key pathogens in oral diseases, susceptibility or resistance to antiseptic agents, and documented associations with systemic conditions. This focused approach aims to highlight microorganisms with clinical relevance to both oral and systemic health outcomes.

## 3. Results

### 3.1. Selection of Sources of Evidence

A total of 629 records were identified through database searching. After removing duplicates and screening titles and abstracts, 12 studies met the inclusion criteria and were included in the full-text analysis.

### 3.2. Characteristics of Included Studies

The included studies were published between 1988 and 2024 and comprised the following: six randomized controlled trials (RCTs), one crossover experimental study, three in vitro experimental studies, and one in vivo experimental comparative study. The included articles examined commonly used antiseptic agents such as chlorhexidine, essential oils, and cetylpyridinium chloride. The main characteristics of the included studies are detailed in Table 1.

### 3.3. Summary of Findings

Across the included studies, antiseptic formulations demonstrated efficacy in reducing pathogenic oral microorganisms and controlling oral diseases like gingivitis and periodontitis. However, several studies also reported alterations in the commensal oral microbiota, suggesting a potential risk of dysbiosis associated with indiscriminate antiseptic use.

## 4. Discussion

This scoping review aimed to systematically map the available evidence regarding the effects of antiseptic formulations on the composition of the oral microbiota and their potential systemic health implications. The findings revealed that while antiseptics such as chlorhexidine, essential oils, and cetylpyridinium chloride are effective in reducing pathogenic oral microorganisms and improving periodontal indices, several studies also highlighted their capacity to disrupt commensal microbial communities, potentially leading to oral dysbiosis and systemic inflammatory effects.

The oral microbiome is a highly individualized, yet functionally stable ecosystem established early in life and influenced by host genetics, the environment, and immune responses [27,28,29,30,31]. Despite taxonomic variation, dominant bacterial phyla—*Firmicutes*, *Proteobacteria*, *Actinobacteria*, *Bacteroidetes*, and *Fusobacteria*—are consistently present across healthy individuals. Commensals contribute to immune homeostasis, colonization resistance, nitrate metabolism, and nitric oxide production [32,33]. The oral environment also includes fungi (e.g., *Candida* and *Aspergillus*) and viruses, especially bacteriophages, which demonstrate host-specific patterns and potential therapeutic use due to their lytic activity [34,35,36]. Disruption of this microbial balance, or dysbiosis, is central to oral diseases such as periodontitis, caries, candidiasis, and oral cancer [37,38,39]. Importantly, disease pathogenesis now emphasizes microbial shifts over specific pathogens. Broad-spectrum antiseptics like chlorhexidine and essential oils may non-selectively eliminate both pathogenic and beneficial microbes, contributing to dysbiosis and antimicrobial resistance [40,41,42,43,44]. Caries-associated microbes include *Actinomyces*, *Lactobacillus*, *Neisseria*, *Prevotella*, and *Candida albicans*, while periodontitis involves red-complex species (*P. gingivalis*, *T. forsythia*, and *T. denticola*) as well as *A. actinomycetemcomitans*, *F. alocis*, and *S. sputigena* [45,46,47]. Disruption of commensals may hinder periodontal tissue regeneration and play a role in oral cancer, periapical periodontitis, and recurrent ulcers, with pathogens like *F. nucleatum*, *P. intermedia*, and *Leptotrichia* implicated [37,46,48,49,50]. Furthermore, oral dysbiosis may affect systemic health through pro-inflammatory mediators (e.g., IL-6 and TNF-α), transient bacteremia, immune cross-reactivity, and microbial translocation (e.g., LPS), contributing to diseases such as cardiovascular and metabolic disorders via the oral–gut–systemic axis [41,51,52,53].

### 4.1. Antiseptic Formulations: Common Agents, Characteristics, and Microbial Impact

Antiseptic formulations are integral to professional dental care and daily oral hygiene, commonly found in mouthwashes, toothpaste, and gels. Their widespread use highlights the need to balance antimicrobial efficacy with safety and potential systemic implications [54,55,56,57,58,59,60]. Table 2 summarizes key agents and their characteristics.

**Figure 2:**
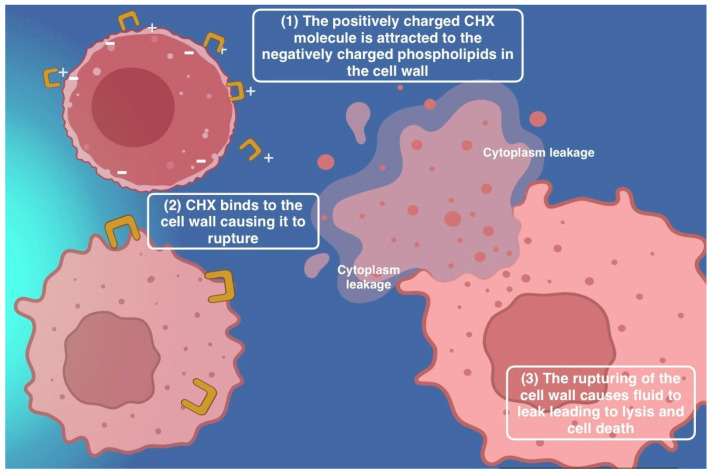
Chlorhexidine (CHX) mechanism of action. Created in BioRender. Jovanova, E. (2025) https://BioRender.com/cw768kp.

Among them, chlorhexidine (CHX) remains the gold standard due to its broad-spectrum action and proven efficacy in reducing plaque and gingival inflammation [62,63,71,72]. However, cytotoxic effects on fibroblasts and osteoblasts, particularly after surgery, necessitate cautious application regarding the dose and timing [71,73,74,75]. Staining and taste alterations further limit long-term compliance [15,16,17,61,76,77]. CHX’s mechanism, which disrupts bacterial membranes, is illustrated in Figure 2 [62,78,79,80,81,82].

Cetylpyridinium chloride (CPC) presents a viable alternative with broader safety margins and demonstrated antimicrobial and antiviral potential, though its antiviral efficacy warrants further research [18,19,20,54,64,83,84]. CPC-CHX combinations may enhance antimicrobial activity while allowing reduced CHX dosage. Natural agents like essential oils (EOs) and propolis offer antimicrobial, anti-inflammatory, and antioxidant benefits but pose risks of hypersensitivity and toxicity, requiring careful formulation and patient guidance [21,22,66,85,86,87,88]. Other antiseptics—including povidone-iodine, hydrogen peroxide, chitosan, and triclosan—vary in their mechanisms and limitations. Povidone-iodine has strong antimicrobial and anti-inflammatory properties but is less commonly used due to staining and taste issues. Hydrogen peroxide is effective against certain pathogens but is limited against biofilms. Chitosan, valued for its biocompatibility and wound-healing properties, still lacks a fully understood antimicrobial mechanism [23,24,25,67,89,90,91,92,93,94,95]. Triclosan, though effective, is increasingly scrutinized due to potential endocrine and neurotoxic risks [96,97,98,99,100]. Overall, antiseptic use must weigh the clinical benefits against safety and tolerability, emphasizing the need for long-term studies, synergistic combinations, and novel formulations using natural compounds. Patient education on appropriate use remains essential [8,26,101,102].

Effective antiseptics must preserve oral tissue integrity, be non-allergenic, and minimize systemic absorption [103]. Table 3 summarizes how antiseptics affect key oral bacteria and their relevance to systemic disease. Beneficial genera such as *Actinomyces*, *Haemophilus*, *Neisseria*, and *Veillonella* aid in converting dietary nitrate to nitric oxide (NO), vital for vascular health [104]. CHX can alter this balance by reducing microbial diversity and nitrite levels, potentially impairing NO-mediated vascular function [105,106]. Short-term CHX use increases *Neisseria* and *Streptococcus* but reduces *Actinomyces*, while longer exposure causes broader depletion. CPC reduces gingivitis-associated microbes with minimal disruption to nitrate-reducing bacteria. EOs and povidone-iodine are less disruptive in this regard, although undiluted EOs may irritate the mucosa and disturb homeostasis [106,107,108].

Microbial resistance remains a concern. CHX resistance involves efflux pumps and membrane modifications [7,104], while CPC resistance arises through efflux, enzymatic degradation, and oxidative-stress-induced mutations [105]. Sublethal QAC exposure may trigger DNA mutations and horizontal gene transfer, promoting cross-resistance and adaptation across microbial communities [105,109].

### 4.2. Systemic Impact of Oral Antiseptics and Related Systemic Diseases

Beyond local antimicrobial effects, oral antiseptics can impact systemic health by disrupting the oral microbiota, influencing vascular function, metabolism, immune responses, and disease susceptibility [12]. Prolonged chlorhexidine (CHX) use has been linked to gut microbiome alterations, reduced nutrient absorption, and impaired metabolism.

CHX can interfere with nitrate-reducing oral bacteria, lowering nitric oxide (NO) availability and elevating blood pressure, with possible implications for metabolic syndrome and diabetes development [110,111,112,113,114]. In contrast, essential oils and povidone-iodine (PVP-I) appear less disruptive to NO-related pathways [23,115,116]. PVP-I use may result in systemic iodine absorption, temporarily affecting thyroid-stimulating hormone (TSH) levels [68,117,118].

Concerns also exist regarding long-term antiseptic use and respiratory health; for example, CHX-induced microbiome alterations may impact chronic obstructive pulmonary disease (COPD) patients. Meta-analyses have suggested associations between frequent mouthwash use and elevated risks of upper aerodigestive tract cancers, including oral cancer, though the epidemiological evidence remains inconclusive [119,120,121].

**Figure 3:**
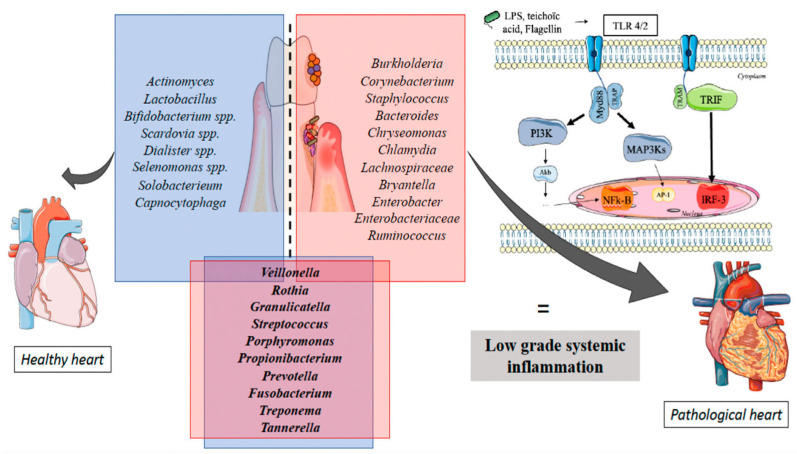
Overview of various bacteria and their roles in triggering low-grade systemic inflammation that contributes to heart disease; reproduced from [123]. Licensed under CC BY 4.0.

Cardiovascular diseases (CVD) are closely linked to oral dysbiosis. Pathogens like *P. gingivalis*, *A. actinomycetemcomitans*, and *Veillonella* have been identified in atherosclerotic plaques [122], while *Lactobacillus* and *Streptococcus sanguis* are associated with endocarditis [123]. Antiseptic-induced disruption of the oral microbiota can enhance systemic inflammation via cytokine release (IL-1β, IL-6, and TNF-α), promoting vascular damage [124,125]. Elevated CRP levels and biomarkers such as Galectin-3 and suPAR further support the link between oral inflammation and cardiovascular risk [125,126,127,128]. Dysbiotic taxa like *Staphylococcus* and *Ruminococcus* contribute to chronic systemic inflammation, reinforcing this association. As shown in Figure 3, specific bacteria in dysbiotic oral microbiota can contribute to low-grade systemic inflammation linked to increased CVD risk [123].

Antiseptic-induced dysbiosis has also been linked to increased risk of respiratory complications. Alterations in the oral microbiome can facilitate aspiration of pathogens, contributing to pneumonia, COPD exacerbations, and COVID-19 severity [129,130,131]. Common respiratory pathogens such as *P. gingivalis*, *F. nucleatum*, and *Prevotella oralis* have been detected in lung aspirates [132]. In immunocompromised patients, such as those with HIV, microbial shifts are associated with systemic inflammation and reduced lung function [133]. Antiseptic overuse may also promote colonization by antibiotic-resistant species, raising concern for respiratory health [134].

Oral dysbiosis has also been implicated in adverse pregnancy outcomes (APOs), including preterm birth, low birth weight, and preeclampsia. Pregnancy-associated hormonal changes alter oral microbial profiles, increasing susceptibility to gingival inflammation [135,136]. Periodontal pathogens such as *P. gingivalis*, *F. nucleatum*, and *A. actinomycetemcomitans* may translocate to the placenta, triggering inflammatory cascades that lead to fetal complications. Elevated levels of *Prevotella intermedia* have been noted in women with preeclampsia. Although some studies suggest CHX mouthwash may reduce APO risk, the evidence remains inconclusive, and further research is needed to clarify safety during pregnancy [12,137].

### 4.3. Alternative Treatments and Future Strategies for Oral Dysbiosis Management

Oral antiseptics are widely used to prevent plaque accumulation and manage oral diseases; however, their overuse—especially chlorhexidine (CHX)—raises concerns about microbial resistance, mucosal irritation, and systemic effects, including disruptions in nitric oxide metabolism, elevated blood pressure, and gut dysbiosis [8,12]. Associations with cardiovascular complications, respiratory infections, and adverse pregnancy outcomes have also been noted [110,134,138]. As such, antiseptics should be used judiciously and tailored to individual clinical needs. Long-term use may benefit from alternatives like essential oils or povidone-iodine, which show lower systemic impact [23,65].

Due to the limitations of traditional antiseptics, alternative microbiome-supportive approaches have gained traction. Probiotics—live microorganisms such as *Lactobacillus reuteri*, *L. rhamnosus*, and *L. acidophilus*—can reduce pathogenic load and improve periodontal and caries outcomes [139,140]. Their mechanisms include modulating immune responses, limiting microbial adhesion, and producing antimicrobial substances [141]. Some probiotics have shown antimicrobial effects comparable to CHX and may offer safer long-term options as summarized in Table 4 [142,143].

Prebiotics, including non-digestible compounds like N-acetyl-mannosamine and beta-methyl-D-galactoside, foster the growth of beneficial bacteria and suppress oral pathogens [144,145,146,147]. Dietary nitrates also function as prebiotics, supporting nitrate-reducing genera such as *Rothia* and *Neisseria.* These agents contribute to anti-inflammatory metabolite production (e.g., SCFAs), promoting microbial balance and mucosal integrity [146,148,149].

Postbiotics—metabolic byproducts or non-viable microbial components—offer benefits without the risks of live bacteria. They can suppress periodontal pathogens, regulate oral pH, produce bacteriocins, and enhance SCFA levels [150], though more human and animal studies are needed [151,152,153].

Quorum-sensing inhibitors (QSIs) represent another novel avenue by targeting bacterial communication pathways rather than directly killing microbes. Natural QSIs derived from plants, algae, fungi, and enzymatic sources—such as lactonases or acylases—disrupt pathogenic biofilm formation while preserving microbial diversity [154,155,156,157,158]. Although preclinical results are promising, further clinical trials are required.

Personalized oral care strategies grounded in microbiome profiling and genomics hold transformative potential for oral disease management. By identifying individual dysbiotic patterns, clinicians can implement targeted interventions—such as customized antiseptic or probiotic therapies and host-modulating agents—for patients colonized by high-risk pathogens like *P. gingivalis* or *T. forsythia* [159,160]. Although the implementation of such precision approaches faces challenges—including the complexity of microbial ecosystems, ethical considerations, and regulatory constraints—they may significantly advance dentistry toward proactive, preventive care [161]. To fully realize this potential, future research must validate microbiome-based diagnostic tools, develop AI-driven systems for early dysbiosis detection and risk prediction, and optimize next-generation probiotics for targeted delivery and improved efficacy. Incorporating host-specific factors such as genetics, dietary habits, and lifestyle will be essential in designing personalized, effective interventions. In parallel, public health policies should evolve in step with scientific advancements by promoting responsible antiseptic use and raising awareness of the associated systemic risks. Coordinated educational initiatives and interdisciplinary collaboration will be critical to reducing disease burden and enhancing oral–systemic health at the population level.

## 5. Conclusions

This scoping review highlights that while oral antiseptic formulations remain essential for managing oral biofilm and preventing oral diseases, their broad-spectrum antimicrobial action can disrupt the oral microbiota’s balance and potentially impact systemic health. The evidence indicates that indiscriminate or prolonged use of antiseptics may contribute to oral dysbiosis associated with systemic conditions such as cardiovascular diseases, respiratory infections, and adverse pregnancy outcomes. Emerging microbiome-friendly alternatives—including probiotics, prebiotics, postbiotics, and quorum-sensing inhibitors—show promise for preserving beneficial oral microbes while controlling pathogens. Personalized oral hygiene strategies, informed by individual microbiome profiles and systemic risk factors, represent a path forward in dental care. However, significant gaps remain in understanding the long-term systemic effects of antiseptics and the clinical efficacy of alternative approaches. Future research should focus on validating microbiome-based risk assessments, optimizing targeted interventions, and integrating multidisciplinary perspectives. Overall, incorporating microbiome-conscious strategies into oral healthcare may improve both oral and systemic health outcomes, emphasizing the oral cavity’s critical role as a gateway to overall well-being.

## Figures and Tables

**Table 1:** Characteristics and outcomes of most relevant included studies of this review.

Study (Author, Year)	Aim of the Study	Study Design	Antiseptic Formulation	Outcomes Measured	Conclusions
Haydari M. et al., 2017 [15]	To evaluate the effect of antiseptic mouth rinses on plaque and gingivitis.	Double-blinded, randomized controlled trial (RCT)	Chlorhexidine	Reduction in plaque and gingivitis scores.	0.2% CHX mouthwash showed a statistically significant superior effect in preventing dental plaque compared to the 0.12% and 0.06% solutions.
Najafi M. H. et al., 2012 [16]	Assess efficacy of chlorhexidine mouthwashes on gingival indices and level of dental staining.	Randomized controlled trial (RCT)	Chlorhexidine	Significant plaque reduction, gingival, bleeding, and stain index.	Lower concentrations of chlorhexidine are recommended to minimize side effects as higher concentrations do not provide additional benefits in controlling plaque and gingivitis.
Cousido M. C. et al, 2010 [17]	To evaluate and compare the in vivo antimicrobial activity of 0.12% and 0.2% chlorhexidine on salivary flora up to 7 h after application.	Experimental, in vivo, comparative study	Chlorhexidine digluconate	Percentage of bacterial vitality in saliva over time using epifluorescence microscopy with SYTO 9/propidium iodide staining.	The 0.2% chlorhexidine mouthrinses showed greater and longer-lasting antimicrobial activity than 0.12%, with double rinsing at 0.2% further reducing bacterial vitality. Concentration influences antimicrobial effectiveness and substantivity.
Teng F. et al., 2016 [18]	To investigate how cetylpyridinium chloride (CPC)-containing oral rinses affect supragingival plaque microbiota and gingivitis progression in humans.	Double-blinded, randomized controlled trial (RCT)	Cetylpyridinium chloride	Changes in supragingival plaque microbiota composition, gingival inflammation, and bacterial network connectivity.	CPC rinses slowed gingival inflammation progression by inhibiting gingivitis-associated bacteria, preserved healthy plaque biodiversity, and disrupted bacterial network connectivity.
Hu D. et al., 2009 [19]	To compare the effects of a 0.05% cetylpyridinium chloride (CPC) mouth rinse versus a fluoride mouth rinse on anaerobic bacteria in supragingival plaque.	Randomized controlled trial (RCT)	Cetylpyridinium chloride	Reduction in anaerobic bacteria in supragingival plaque	CPC mouth rinse significantly reduced anaerobic bacteria in plaque more than fluoride rinse after both one use and 14 days, with no adverse events reported.
Rioboo M. et al., 2012 [20]	To evaluate the effects of a mouth rinse and dentifrice containing 0.05% CPC on plaque and gingivitis in patients with gingivitis.	Double-blind, parallel, randomized clinical trial	Mouth rinse and dentifrice with 0.05% cetylpyridinium chloride	Plaque index, gingival index, patient-based and microbiological variables.	Limited benefit of CPC formulations in reducing plaque and no significant effect on gingivitis as adjuncts to unsupervised oral hygiene.
Karbach J. et al., 2015 [21]	To compare the in vitro antibacterial activity of various essential oils versus standard oral antiseptics against oral microorganisms.	In vitro antimicrobial study	Essential oils tested: tea tree oil, eucalyptus oil, lemongrass oil, eucalyptus-based mixture compared with chlorhexidine digluconate, povidone-iodine, and octenidine dihydrochloride	Size of antimicrobial inhibition zones against oral bacteria and *Candida* species.	Some essential oils, especially lemongrass oil, showed stronger antimicrobial effects than standard antiseptics, suggesting potential for clinical and oral hygiene use. Further research needed on concentrations and application methods.
Nikolić M. et al., 2016 [22]	To investigate the chemical composition, antimicrobial activity, and cytotoxicity of commercial essential oils from *Hyssopus officinalis*, *Rosmarinus officinalis*, and *Salvia officinalis* against oral pathogens.	In vitro experimental study	Essential oils from *H. officinalis*, *R. officinalis*, and *S. officinalis*	Chemical composition, antimicrobial activity against oral *Candida* spp. and bacteria; cytotoxic potential.	All tested essential oils were active against oral pathogens, with *S. officinalis* oil showing the lowest antimicrobial activity. The oils show promise as natural agents for preventing or treating oral diseases, though careful formulation is needed.
Mitsui T. et al., 2017 [23]	To evaluate the effects of different mouthwashes on salivary nitrate/nitrite levels and oral nitrate-reducing bacteria after nitrate intake.	Crossover experimental study	Essential oil mouthwash, povidone-iodine, chlorhexidine, and water (control)	Salivary nitrate/nitrite levels (colorimetric assay) and presence of *Veillonella dispar* at 0, 1, 5, and 10 h post-treatment.	Chlorhexidine significantly reduced *V. dispar* presence, suggesting potential inhibition of nitrate-reducing activity with repeated use. Essential oil and povidone-iodine had minimal effects.
Gusberti F. A. et al., 1988 [24]	To compare the clinical and microbiological effects of 0.12% chlorhexidine (CHX) and 1% hydrogen peroxide (H_2_O_2_) mouthrinses in an experimental gingivitis model.	Randomized controlled trial	0.12% chlorhexidine mouthrinse and 1% hydrogen peroxide mouthrinse	Gingivitis incidence, bleeding sites, plaque scores; microbiological composition of supragingival and marginal plaque.	CHX was highly effective in reducing gingivitis, bleeding, and plaque, and significantly reduced a wide range of oral bacteria. H_2_O_2_ had minimal clinical or microbiological benefits.
Oliveira M. S. et al., 2024 [25]	To evaluate the antimicrobial effects of tea tree oil and chitosan alone, and in combination, against oral pathogens and biofilms, and to assess the chemical composition and cytotoxicity of TTO.	In vitro experimental study	Tea tree oil, chitosan, and their combination	Antimicrobial activity, biofilm inhibition, synergy, chemical composition of TTO, bacterial growth delay, and fibroblast, cytotoxicity.	TTO and CH showed effective antimicrobial and antibiofilm activity against oral pathogens. Their combination was synergistic and non-cytotoxic at tested concentrations, offering a potential natural strategy against antimicrobial resistance.
Dehghani M. et al., 2019 [26]	To evaluate and compare the effects of propolis and chlorhexidine mouthwashes on plaque, gingival, and periodontal indices in patients undergoing fixed orthodontic treatment.	Triple-blind, randomized clinical trial	Propolis mouthwash and chlorhexidine mouthwash	Plaque index, gingival index, and Community Periodontal Index measured before and after 3 weeks of mouthwash use.	Both propolis and chlorhexidine mouthwashes significantly improved PI, GI, and CPI. Propolis showed similar effectiveness and may be a suitable alternative to CHX without its side effects.

**Table 2:** Major characteristics of the most commonly used oral antiseptics.

Oral Antiseptics	Most Common Formulations	Spectrum	References
Chlorhexidine	Oral rinses, aerosols, and spray formulations (0.12–0.2%) Gels (0.12–1%) Dental varnishes (1%, 10%, 40%) Toothpaste, gels for cleaning teeth, and dental flosses	Broad activity: stronger against Gram-positive bacteria and less effective against Gram-negative bacteria. Also active against fungi and some lipophilic viruses	[61,62,63]
Cetylpyridinium chloride	Mouthrinses and toothpaste: 0.05–0.10%	Broad antimicrobial spectrum: most effective against gram-positive pathogens and yeast	[18,54,64]
Essential oils	Primarily applied externally (e.g., in mouthwashes) for maximum effectiveness	Broad spectrum of antibacterial, antifungal, antiviral and insecticidal fungi and yeast; in addition, potential to inhibit the growth of drug-resistant microbial strains and antioxidant and anti- inflammatory properties	[65,66]
Povidone-Iodine	Local topical solution (7.5%, 10%), spray (5%), Povidone-iodine solution Fe-150	Broad antibacterial spectrum: Gram-positive and Gram-negative; bacteria spores, fungi, protozoa, and viruses	[67,68]
Triclosan	Toothpaste and mouthrinses 0.3%	Broad antimicrobial action against Gram-positive and Gram-negative bacteria and fungi	[69,70]

**Table 3:** Commensal oral bacteria and their role in systemic diseases.

Bacteria	Commensal (Healthy State)	Dysbiosis (Associated Systemic Diseases)
*Porphyromonas gingivalis*	Present in low levels in healthy oral microbiota	Strongly associated with CVD, Alzheimer’s disease, and periodontitis
*Fusobacterium nucleatum*	Part of normal oral flora, involved in periodontal health	Linked to CVD, adverse pregnancy outcomes (preterm birth, low birth weight), and colorectal cancer
*Streptococcus* spp.	Predominant in healthy biofilm, help in plaque formation	Overgrowth associated with CVD, diabetes, and periodontal disease
*Prevotella* spp.	Present in low abundance, contribute to balance	Overgrowth associated with periodontal disease, diabetes, and adverse pregnancy outcomes
*Lactobacillus* spp.	Contribute to oral health, maintain pH balance	Overgrowth linked to dental caries, potentially contributing to CVD through inflammation
*Neisseria* spp.	Part of healthy oral microbiota, aid in microbial balance	Dysbiosis may contribute to respiratory infections and periodontal disease
*Actinomyces* spp.	Present in healthy individuals, play a role in homeostasis	Associated with periodontal disease and CVD through inflammation
*Veillonella* spp.	Maintain oral balance in healthy individuals	Dysbiosis linked to periodontal disease, may exacerbate CVD due to inflammatory response
*Tannerella forsythia*	Present in healthy oral microbiome in low abundance	Overgrowth linked to periodontitis, CVD, and Alzheimer’s disease through systemic inflammation

**Table 4:** Direct and indirect mechanisms of probiotics in oral health.

Probiotics: Mechanism Type	Description
Direct Mechanisms	- Action on plaque formation by competing with bacteria-to-bacteria attachments - Competing with oral microorganisms for substrates available - Production of antimicrobial substances that inhibit oral bacteria - Involvement in the binding of oral microorganisms to proteins [143].
Indirect Mechanisms	- Effect on local immunity and non-immunologic defense mechanisms - Regulation of mucosal permeability - Modulating systemic immune function - Oral colonization by less pathogenic species [143].

## Data Availability

The original contributions presented in this study are included in the article/Supplementary Materials. Further inquiries can be directed to the corresponding author.

## Supplementary Materials

The following supporting information can be downloaded at https://www.mdpi.com/article/10.3390/antibiotics14080815/s1. Preferred Reporting Items for Systematic reviews and Meta-Analyses extension for Scoping Reviews (PRISMA-ScR) Checklist. Reference [162] is cited in Supplementary Materials.
